# New Elements of Operative Surgery

**Published:** 1846-06-01

**Authors:** 


ART. III.—*New Elements of Operative Surgery.* By ALF. A. L. M. VELPEAU, with a Treatise on Minor Surgery, illustrated by 300 Engravings; and an Atlas in quarto of 22 Plates. First American, from the last Paris edition. Translated by P. S. Townsend, M. D.: Augmented by several hundred pages of new matter, under the supervision of, and *with Notes and Observations by Valentine Mott, M. D.* In three volumes, 8vo. Henry G. Langley, New-York, 1846.

The second volume of this great work has been received, and the concluding volume is announced for October, 1846. Already does the work comprise over 2000 pages of closely-printed matter, and when completed, it will exceed 3000—the largest surgical book, we believe, ever issued from the American press. Whether it possesses any other quality of excellence than size, we shall see.

M. Velpeau is the most indefatigable and reliable medical *compiler* in Europe, to whatever department of the science he may for the time direct his attention. For all, therefore, that pertains to the "History" of surgery he was qualified, and we are now prepared to say, having for some time been possessed of the *complete* original work, that our author has herein left little to be supplied. And for this, at least, he deserves our thanks, since he has "vindicated the silent and unprotected dead, and rebuked the ambitious living" not only, but he has erected also an archive, whose records of unsuccessful as well as successful experiments, will guard those who are prudent enough to consult it, against wasting their time in similar fruitless adventures.

But M. Velpeau possessed for this enterprise other special qualifications. He is a most accomplished anatomist; and in a knowledge of the "principles" of surgery, he has no superior; nor, indeed, in a theoretical acquaintance with all the manual details of operative surgery. As a practical operator, however, his experience is certainly second to that of Roux or Lisfranc. But then, when we remember that for twenty-three years he has been teaching anatomy or surgery, and during a greater part of this time has had charge of surgical wards in such establishments as St. Antoine, La Pitie, and La Charite, we cannot but see that he must have tested well and often most of the operations which he describes.

Fortunately however, if we must in fairness admit that, as a practical operator, our author does not bring as ample qualification, as certainly he possesses in all other points, yet this very deficiency would seem to be most aptly and appropriately supplied by the American annotator, Dr. Mott. And we have thought that a duumvirate could scarcely have been found better fitted by the great but different talents of the associates to erect a complete and harmonious "surgical edifice." In the connection, therefore, of Dr. Mott with this American edition of Velpeau, we have but one thing to regret, and that is, that the announcement of the title page is not *literally* sustained, and that instead of "Notes and Observations by Valentine Mott, M. D.," we have, with I believe not an exception, "Notes and Observations" by P. S. Townsend, M. D., *endorsed* by Dr. Mott! It may really make little difference as to the value of the matter thus communicated, and we may be thought over-exact in our requisitions; but when Dr. Mott, in addition to the announcement in the title page, declares also in a letter to Velpeau himself, "I have taken the liberty to enter my name as a *compagnon de voyage* with you," we had every reason to suppose that the seat which he had taken beside the distinguished "voyageur" was to be occupied by himself, and not by his "Boswell."* And however interesting and instructive, the intimate acquaintance which this gentleman possesses of his patrons' life and doings, may render his conversations, yet we doubt whether Velpeau will feel himself complimented by the substitution. And without intending to question the competency of Dr. Townsend, we must be allowed to declare the disappointment which we, in common no doubt with the whole medical public, have felt, that we have not in these notes the "handwriting and signature" of our own beloved Mott. To speak plainly, it is a *deception*, for which some one will be held responsible.

But to the book—and we shall at present look only at the first volume; which has already been before the public for a whole year.

"*Hygienic Precautions in relation to Operations.* Other things being equal, if the spring and autumn suit better than the winter or midsummer, it is not only because their temperature is more mild, but also because the system is then in a better condition to resist general morbid reactions. So also we should not perform, except in temperate seasons, lithotomy," &c. &c. (p. 9.)

The above corresponds with the views which we have always entertained

* "I felt somewhat hurt at this comparison, and, I believe Dr. Pozz perceived it, for as he was squeezing a lemon he said, 'Never be affronted at a comparison, I have been compared to many things myself, but I never was affronted. No, sir, if they would call ME a dog, and YOU a cannister tied to my tail, I would not be affronted.'"

"How to Write the Life of a Friend."

upon this matter, and is equally in accordance with the opinion of Dr. Gibson, of Philadelphia; who, in reply to M. Velpeau, (p. 45 of appendix) ascribes to the more temperate and equable climate of his own city, a superior success in surgical operations, compared with results in the larger cities of Europe, such as London, Paris, &c.

From all this, however, Dr. Mott (by Townsend) differs *toto-coelo.* Denying first, the mildness or uniformity of "our" climate as compared with that of Europe. And in this he has the right beyond doubt; nor do we understand Dr. Gibson to have affirmed the contrary, since he spoke only of the climate of Philadelphia. And second, "the experience of Dr. Mott" says *Dr. Townsend*, "both in this country, and in Europe, Asia and Africa, is directly to the point that it is precisely during those extreme and arid summer heats, which Prof. Gibson regards as unfavorable, and when the thermometer is ranging in the neighborhood of 100° Fahrenheit! that surgical operations always do better than at any other time." And *Dr. Townsend* farther adds "the same benefical results which an elevated and dry temperature produces upon the processes of adhesive inflammation, seem to be derived also, says Dr. Mott, from the tonic power of intense cold, during our protracted winters," (p. 46, appen.).

Whether in this as well as in the affair of aneurisms (p. 47, appen.) Dr. Mott and his mouth piece, have been influenced by no kindly feeling towards their eminent countryman, or whether they have actually convinced themselves that intense heat and intense cold are more sanative than a mild and uniform temperature, we have not assured ourselves: but of one thing we are certain, and we affirm it broadly without fear of its disproval, that so far as climate or temperature have any influence, it is favorable or unfavorable in exact proportion as it is temperate or extreme, uniform or variable: and that whatever Dr. Mott may have learned in "Europe, Asia and Africa," he will never teach us to depress the temperature of a surgical ward below zero, or on the other hand, to elevate the same to 100° Fahrenheit,* nor indeed to select the extreme of winter's cold, or summer's heat, for the performance of a capital operation.

The remarks which follow in this chapter are highly valuable. Some of them may be placed in the form of aphorisms, and we shall do well to remember them.

"With infants we need not be in haste to operate for lacrymal tumors, hydrocele, enlarged tonsils, &c., because the growth of the individual often

*An "ingenious young physician" M. Jules Guyot, has proposed to hasten the cicatrization of wounds, by enclosing the limb or part injured in an air-tight stove, and elevating the temperature of the contained air to from 80° to 122°!

causes these diseases to disappear. We desist often from the operation for hydrocele, cataract, artificial pupil, &c., in old people, because they are less likely to succeed, and when most successful they are less serviceable, " etc. (p. 11,).

"For operations choose the morning," the patient is then generally "less fatigued," and more of the day remains to provide against accidents. To which we will add, the light is usually better in the morning than afternoon, a point of no mean consideration in the performance of many operations.

"As a general rule, operations with the day and hour fixed, which Pouteau compares to a species of *auto-da-fe*, are bad." This rule admits of many exceptions.

Celsus has said "Melius anceps remedium quam nullum," but M. Velpeau would say "Mieux vaut laisser mourrir un malade que de le tuer," (p. 14) and so say we, for we could never countenance those two edged remedies which are said always to either "kill or cure": and we doubt whether many surgeons or physicians could be found, whose patients having died under the "remedium anceps" would confess the murder. We do not deny that certain hazards may be justly incurred, nay, that all operations are attended with some hazard and uncertainty, but it is that reckless principle which has led to most of the operations for the removal by excision, of ovarian tumors, of degenerate thyroid glands, of the uterus in situ, &c., &c., which M. Velpeau would discountenance and reprove.

The old axiom "*sublata causa, tollitur effectus*" will not always apply to the removal of a diseased part. Other parts in the neighborhood, or the whole system may have become so far tainted that the extirpation of the original offence shall fail to eradicate the disease.

"Almost all the preparations, whether hygienic or medicinal, to which patients were formerly subjected before being operated upon, have been abandoned by the moderns. These preparatory steps are nothing in fact," says Pouteau, "but a protracted meditation upon the malady." * * * "Nevertheless there are some of them that deserve to be retained when the nature of the lesions allows of delay." (p. 15.)

Our own great surgeon, Dr. Physic, used to say that whatever of superior success may have attended his surgical operations, ought to be ascribed mainly to the more ample preparation to which he always submitted his patients. Dr. Dudley, of Lexington, the unrivalled lithotomist, attributes also his astonishing success in this class of operations to the same course of preparation: and while we accord to both Physic and

Dudley, with many others, all they claim in favor of *judicious* preparation, we cannot conceal our satisfaction at the guarded manner in which M. Velpeau evidently intends to sanction certain preparatives. Thus, bleeding, catharsis, dieting, &c., are sometimes necessary, but employed indiscriminately they are often eminently hurtful, from the irritable, nervous, and prostrated condition which they so frequently induce.

"*To prevent pain during an operation.*" M. Velpeau has lived in the midst of the most marvelous manifestations of the powers of *Mesmerism:* he has witnessed its public and private exhibitions; again and again he has heard it discussed in the "Royal Academy of Medicine," of which he is a member, and enjoyed a thousand other opportunities of learning its value, or rather we should say, its existence. His opinion is then worth recording.

"Nor has magnetism been forgotten. All the journals have rung with the account of an extirpation of the breast, without the patient being conscious of it," &c. "But everything leads to the belief that in such cases the operators were deceived by the (natural) insensibility, or the chicanery of the patients, or by some confederate," (p. 23). * * * * "A peasant amputated his own limb with a coarse saw, according to Scharsmidt. I have amputated the thigh of three patients, who did not utter the slightest cry during the operation. A robust man, otherwise very susceptible, chatted tranquilly while I was extirpating a large sarcocele, without manifesting the least sign of pain."

In regard to the claims of this so-called "science," we have had occasion to know, that nearly all the medical savans of Paris as well as London, bear similar testimony. And as to the ability of very many patients to endure operations of great severity, without complaint or evincing the slightest indication of pain, we have had in our own practice frequent and most striking demonstrations, where no aid from Mesmerism had been invoked.

To conclude, our author remarks that "To avoid pain in operations, is a chimera that we can no longer pursue in our times." For neither opium nor lettuce, hellebore nor rue, are found any more capable of rendering a nerve insensible to the amputating knife, or to "lenify the pain" than the infusions of Iapis, or the sympathetic powders of Digby. A steady hand and a keen blade carried "cito, tuto et jucunde" are the only palliatives to which modern surgery resorts.

*Entrance of air into veins,* during operations. This interesting subject is fully and ably discussed by Velpeau. A list of 40 reported cases is presented and their respective claims to credence analyzed, a summary of which is as follows. That air entered the veins is probable in 10 cases—uncertain in 8 cases—extremely probable in 2—almost certain in 3—the balance are not reported in such a manner as to enable him to form an opinion of their value: and he concludes by stating with that philosophic candor, which is so characteristic of himself, "As for myself, I repeat, that this kind of accident appears to me to have been many times met with; only I feel that until there are proofs more conclusive, this opinion cannot be any thing but a personal belief, and that science possesses nothing at the present day which can change this belief into a fixed and general *conviction*." Dr. Mott, however, (through his interpreter) in a note expresses a full conviction that the case communicated by himself to M. Velpeau was sufficiently sustained; but after a careful examination of his own report, we think the reader will say with M. Velpeau, that nothing proves it to have occurred in this case more than in the others which are rejected.

In the section devoted to various modes of arresting hemorrhage we have an enumeration of some of the curious suggestions of surgical experimentalists—such as the compression of the stump with a roller, (without ligatures) by Kock, of Munich—the bruising of M. Briot—the plugging with a "cone of alum or sulphate of copper," (wax is too slippery!)—the probe of Chastenet—the extremity of a bougie, made of catgut, deerskin, or chamois. By "renversement" or doubling the artery upon itself—much like the process of skinning an eel, but somewhat more difficult: by "perpendicular compression," "torsion," &c. &c. Some of which are sufficiently absurd, while others have a grain of reason in their defence, but all of which, since they are santioned by one or more of his "confreres" he feels himself bound, in courtesy, to discuss gravely.

And now that the "Eau Brochieri" is exciting the wonder and admiration of the credulous, as a haemostatic agent, it may not be amiss to mention that other waters than those of the Italian charlatan have been *known* to possess like power; such were the waters of M. Binelli, which would instanter restrain "every kind of hemorrhage," and that of M. Falrich and Grand, "whose efficacy has been placed beyond doubt," not to speak of the assuasive words of Tom Pots the "serving man," who

> "Bound his kerchief on the wound,
> And with some kind words staunched the blood."

Yet after all, surgeons will probably conclude that there is no more effectual way of closing a bleeding artery, than by securing it with a ligature as Liston says, in a "good, honest, devilish tight and hard knot."

Upon the vexed question (vexed only in France) of union by first or second intention, M. Velpeau, in reply to M. Pelletan and Gouraud, who insist upon "secondary union every time we operate for an old disease," holds the following language. "As for myself, I can aver, that if the accidents mentioned by Pelletan often occur, it is much more for a want of sufficient precautions, than as an unavoidable consequence of the operation; allow that there may be some danger in shutting in eight days the wound that results from the removal of a limb, which the constitution had for a long time transformed into a secretory organ; but are not these exceptions? And can reasons so feeble, and for the most part questionable, weigh down against all the perils that we risk by indiscreet union?"

This is remarkable language for a Parisian Surgeon, and indicates that our Gallic friends are making some progress towards civilization in this matter, or rather, perhaps, that the Guizot policy—the royal tete-a-tetes—the exchange of annuals and bagatelles, are extending their influence over medical as well as national politics; and that the English discoverer of "union by first intention" is about to receive that justice at the hands of the French, which their inveterate prejudices have so long withheld from him. And we shall not be surprised to hear soon, that the great surgeon of Leeds has been honored with a niche in the Royal Academy, and his "invention" as the French facetiously term it, secured to themselves by a "patent."

For the plan "invented" by M. Velpeau, for the treatment of erysipelas, by solutions and unguents of sulphate of copper, which he has found so eminently successful, we refer the reader to the author himself, and to the numerous abstracts now to be found in the various American Journals.

"Title second" is devoted to Minor Surgery, and treats in detail of instruments necessary for elementary operations; of bandages, plasters, sutures, splints, dressings for fractures, &c. &c. In relation to splints, Dr. Townsend, by authority of Dr. Mott, makes the following note:

"Felt made into slabs, sheets, &c. often half an inch thick, is much better than pasteboard, and when wet in warm water, or over steam, perfectly soft and flexible. When dry, they are as hard as a board, and harder than pasteboard. They are very cheap and serviceable." (p. 133). We feel bound to add that we are indebted for the introduction of these splints, to Mr. G. T. Warner, who manufactures them upon a large scale at St. Johnsville, N. Y., moulding them with most perfect accuracy to the shape of the different limbs, and putting them up to order in nests, and in sheets. We have for some years past been accustomed to use them in almost every fracture, and we feel warranted in saying that they are greatly superior, not only to pasteboard, but also to the dextrine or starch bandages so much lauded by M. Velpeau.

Listen now to what he says in behalf of dextrine. "Thus have we said all that is necessary in relation to fractures, provided practitoners would imitate what I have done at La Charite, since the month of January, 1837. In every case we perceive, that the bandages saturated with dextrine answer the purpose. When we consider that by this means the patient is enabled to move and turn himself in bed, even to raise himself up, and to walk with crutches from the third or fifth day, it may be asked if hence-forth there will be any need of cushions, splints, fracture boxes, inclined planes, leathers, ties and foot-boards, in the bandages designed for fractures." (p. 190.)

M. Velpeau is an enthusiast in the matter of starch and dextrine, and with Beau Brummel he is ready to exclaim, "Starch, sir, is the man." And really M. V. is, at La Charite, generally very "fortunate"—but neither he nor M. Suetin are yet working anything like a rapid conversion of the world to their favorite plan. And *Dr. Townsend, in behalf* of Dr. Mott, intimates that a counterblast may be found in Dr. Mott's "Recent Book of Travels in Europe and the East."

Abernethy used to delight in teaching the "Big Wigs" at the Royal College of Surgeons how to make a *poultice.* M. Velpeau also does not hesitate to speak of "cataplasms of flaxseed" as applicable to an infinite number of wounds and ulcers, (p 214) and to indicate their time and mode of application, &c. &c. Dr. Mott however, (through Dr. Townsend!) "is fully of opinion that continued poulticing of wounds, after the inflammatory symptoms are reduced, greatly diminishes the vitality and tone of the part, retards or vitiates the granulations" &c.

In "Title third" may be found minute directions for bleeding, cupping, leeching, vesication, for application of issues, setons, the cautery, moxa, and vacinations, also a complete treatise on Dental Surgery. "Title fourth," is miscellaneous and includes a mass of matter not readily embraced under the previous titles — such as acupuncture, sutures, warts, corns, sub-lingual fungosities, anatomy and exsection of cicatrices, diseases and deformities of tendons with the appropriate operations, deformity of bones, and an abridgement of the entire work of M. V. on strabismus published in Paris in 1842 — with nearly one hundred pages upon the various anaplastic operations, and an appendix of nearly two hundred pages, by the publisher, devoted chiefly, to the new operations of such distinguished Americans as, Mettauer, Mutter, Pancoast, Post, Watson, Buck, &c. &c. and finally to a retrospect, or general review of the progress of sub-cutaneous sections and anaplastic Surgery, since the last edition of Velpeau was issued.

For all this we have no space, but we cannot conclude without referring our readers to one page under the section "Strabismus," as indicating something of spirit of the perseverence and experimentalism, which is peculiar to France, and of which our author is but a modest representative; which page will also in itself, be sufficient evidence that just such a missionary as M. Velpeau's maxim, "better let the patient die, than to kill him" is much needed in Paris.

A countryman aged 46, had had both nystagmus and myopia, from infancy. M. Velpeau had long been convinced of the action of the straight muscles of the eye in producing staphyloma, and he now thought they might in this case be the cause of the myopia, and he resolved to attempt its relief. "I practiced, he says, "the section of the internal straight muscle of one side only. I waited twelve days and then cut the external straight muscle, in order to correct a strabismus divergens and a troublesome diplopy, *both of which were already established, in consequence of the operation*"! M. Velpeau now ascertained that the myopy had diminished one half, but the diplopy persisted, to remedy which, he next cut the rectus externus of the opposite eye. At first the myopy in this eye diminished also, but soon vision became vague and confused, and the double vision which for a while had ceased, again returned. The eye first operated upon now began to turn in again, and M. Velpeau cut the rectus internus the second time, the rectus externus having been already divided, the eye would not turn back, and he was obliged to force it out by continued pressure upon the inner side of the ball. The eyes finally became straight, but remained unfortunately almost immoveable! Six months after, our operator again saw his patient, and he frankly tells us that he then had double vision, confused vision and double convergent strabismus—"diplopie tres prononce et une amplyopie incontestable, un legre strabisme convergent l'etait etabli des deux cotes" (p. 170 of original work). Again with the most indomitable perseverance, M. Velpeau divided the internal rectus of both eyes, cutting the stubborn muscles for the sixth time. The eyes now again became straight, but the double vision, the confused vision, the immobility remained — and we will also suggest that if the report had been delayed a few days longer, the return of the strabismus might have also been enumerated among the final results.

After all, although we are not quite satisfied with the book, yet we are as well suited as we ever expect to be—for if it have faults, they are so few, and so generally unimportant, that they do not exceed the just allowance of imperfections which will ever attend all human efforts. Most heartily, therefore, do we congratulate the American medical public on the appearance of this excellent translation of one of the very best works upon surgery yet published, at home or abroad; and in spite of our own peculiar taste, we say, in all sincerity, that we are truly grateful to Dr. Townsend for his numerous and well-selected additions, as well as for his valuable notes, *endorsed by Mott*, and we cheerfully recommend these joint labours to our surgeons, as constituting the most systematic, and so far as we can determine from the volumes before us, the most complete surgical book we have ever read. F. H. H.

